# Aberrant Expression of *miR-103*, *miR-184*, *miR-378*, *miR-497* and *miR-506* in Tumor Tissue from Patients with Oral Squamous Cell Carcinoma Regulates the Clinical Picture of the Patients

**DOI:** 10.31557/APJCP.2020.21.5.1311

**Published:** 2020-05

**Authors:** Maryam Ghaffari, Milad Asadi, Dariush Shanehbandi, Soghra Bornehdeli, Mahsa Sadeghzadeh, Haniye Mohammad Reza Khani, Shahram Ghasembaglou

**Affiliations:** 1 *Tuberculosis and Lung Diseases Research Center, Tabriz University of Medical Sciences, Tabriz, Iran. *; 2 *Immunology Research Center, Tabriz University of Medical Sciences, Tabriz, Iran. *; 3 *Medical Faculty, Tabriz University of Medical Science, Tabriz, Iran. *

**Keywords:** Oral squamous cell carcinoma, miR-103, miR-184, miR-378, miR-497, miR-506

## Abstract

**Background:**

This study aimed to evaluate the expression patterns of *miR-103*, *miR-184*, *miR-378*, *miR497* and in squamous cell carcinoma (SCC) of the tongue and to be compared with normal peripheral tissues.

**Methods:**

Tumor and marginal tissues were obtained from 50 patients with OSCC. After RNA extraction, expression level of *miR-103*, *miR-184*, *miR-378*, *miR497*, and *miR506* was estimated using SYBR green master mix and real-time quantitative PCR.

**Results:**

It was observed that, there was no detectable difference in expression level of *miR-103 *between tumoral and marginal tissues. However, expression level of *miR-184*, and *miR-378* showed significant increase in tumor tissue samples compared to marginal tissue samples. *MiR-497* and *miR-506* demonstrated considerable decrease in tumoral cells in comparison with peripheral tissues. Moreover, the expression level of *miRNAs *was associated with clinicopathological features of the patients.

**Conclusions:**

Our data indicated that *miR-184*,* miR-378*,* miR-497*, and *miR-506* can be used as a potential diagnostic and prognostic biomarker in OSCC. Nevertheless, more studies are needed to confirm this claim.

## Introduction

Oral squamous cell carcinoma (OSCC) is estimated for almost 90% of oral cancers and most typically between people of South-Central Asia (Deng and Liu, 2015; Healy and Moran, 2019). However, quick or early recognition of it is necessary and effective for increasing survival ratio and prognosis. Tobacco, age, using alcohol and betel nut are considered as the most risk factors for causing OSCC (Vonk et al., 2019). Besides, among above-mentioned risk factors, smoking increases the incidence of diseases by approximately 80%, and also based on epidemiological investigations, combination of drinking with smoking leads to a significant increase in rate of OSCC (Sarma et al., 2019; Shree et al., 2019). On the other hand, many patients show the disease without interference of these risk factors, in which the role of poor oral hygiene and tooth loss are prominent reasons associated with OSCC, and it proves that oral bacteria play a role in oral cancer (Siriwardena et al., 2018; Healy and Moran, 2019). Therefore, molecular aspect of OSCC may be a significant alternative to establish pioneering therapeutics method (Markopoulos, 2012). *MiRNAs*, as a prominent gene expression regulator, are short, single-stranded noncoding RNAs, consisting of approximately 18-24 nucleotides in length. First, long and primary form of miRNA, which is encoded in nucleus, is transported in the cytoplasm to be actively processed via cellular nuclease such as Dorsha, and this cytoplasmic miRNA is converted to mature form by means of Dicer Dicer (Hu et al., 2016; Asadi et al., 2018b). Base-pairing to specific region of target genes (3′-untranslated region (3′-UTR)) is the main purpose of mature miRNA by which promotes either mRNA degradation or suppression protein translational process (Chang et al., 2018; Dong and Liu, 2018). MiRNAs with various physiological and pathological activities including growth, cell cycle, apoptosis, development, and differentiation play crucial role in carcinogenesis or tumor progression and suppressive in tumors including OSCC (Soltanzadeh-Yamchi et al., 2018). In addition, function of dysregulated miRNAs, as tumor suppressors or oncogenes, depends on their target mRNAs (Dong and Liu, 2018). Upregulated miRNAs are known as oncogene, which cause negative regulation of tumor suppressor gene; by contrast, tumor suppressor miRNAs, as a downregulated one, function in repression of tumor development (Zhu et al., 2012; Sadeghiyeh et al., 2019). This study investigated aberrantly expressed *miR-103*, *miR-184*, *miR-378*, *miR-497* and *miR-506* in OSCC and evaluated attribution of these miRNAs’ expression with the clinical outcomes of the patients with Iranian Azari society.

## Materials and Methods

*Study Subjects*

This study consists of a total of 50 patients with confirmed tongue cancer (29 men and 21 women) that their peripheral tumor-free tissues were included from Iranian patients of the Azari ethnicity, northwest of Iran with a mean age of 49.25 ± 8.47 years. All patients were histologically confirmed as tongue cell carcinoma and referred to Imam Reza Hospital of Tabriz University of Medical Sciences between 2015-2017. Tumor tissue and marginal tissue, as a control group, were collected during the surgical procedure and then transferred into RNase inhibitor solution (Qiagen, Cat No./ID: 76104) and were immediately stored at −80^o^C until RNA extraction. By collecting samples, we eliminate patients undergoing chemotherapy and radiation therapy. Clinicopathological characteristics of the patients are summarized in Table 1. The study protocol was approved by Local Ethical Committee of Tabriz University of Medical Sciences and written informed consent was obtained through all subjects.

*MiRNA Extraction*

Isolation of total RNA from tumoral and marginal tissues was performed using Tripura isolation reagent (Roche, Cat No.11667165001) according to the manufacturer’s instructions. Concentration and purity of extracted RNA by means of UV spectrophotometer were determined using a NanoDrop at 260/280 nm (NanoDrop ND-2000C Spectrophotometer, Thermo Fisher Scientific, USA). Furthermore, RNA integrity of the samples was examined by 1% agarose gel electrophoresis. Then, RNA samples were stored at – 80 °C until accomplishment of cDNA synthesis (Asadi et al., 2018b).

*Real-Time Quantification of MiRNAs*

Real-time polymerase chain reaction (PCR) was conducted for quantitative measuring the microRNAs expression level. In first step cDNA was synthesized using total RNA and miRNA specific primer according to the Universal cDNA Synthesis Kit (Exiqon Cat No.40023301). In second step quantitative real-time PCR was accomplished by Exilent SYBR Green Master Mix (Exiqon, Cat No. 400203421) and miR-34a specific primer set (Exiqon, Cat No. 400204481). Besides, U6, as an internal control, was applied to normalize expression level of target MicroRNAs. Finally, relative expression level of duplicated samples was analyzed by the comparative threshold cycle (Ct) method as explained by Pfaffl.

*Statistical Analysis*

Statistical analysis was conducted using the GraphPad Prism 6 (GraphPad Software Inc. San Diego, CA, USA). Differences between expression level of CRC tissues and their paired marginal tissues were examined for statistical significance by Student’s t-test. Kolmogorov-Smirnov’s normality test was used to evaluate normality of data. Relationship between expression of target genes and patient’s clinical parameters was evaluated through Pearson’s correlation test. All obtained results were represented as mean ± standard deviation (SD). Statistical significance level for all P value was less than 0.05.

## Results

We evaluated *miR-103*, *miR-184*, *miR-378*, *miR-497 *and *miR-506* expression in 50 patients with tongue cancer samples and 50 paired noncancerous samples. The association of mentioned *miRNAs* expression with clinic-pathological parameters of tongue cancer patients was summarized in Table 1. As a result, expression level of miRNAs was found to be closely correlated with venous invasion and differentiation as two pathological features. However, there were no significant differences regarding age, gender, tumor size, and lymph node metastases in tongue cancer patient.

**Figure 1:**
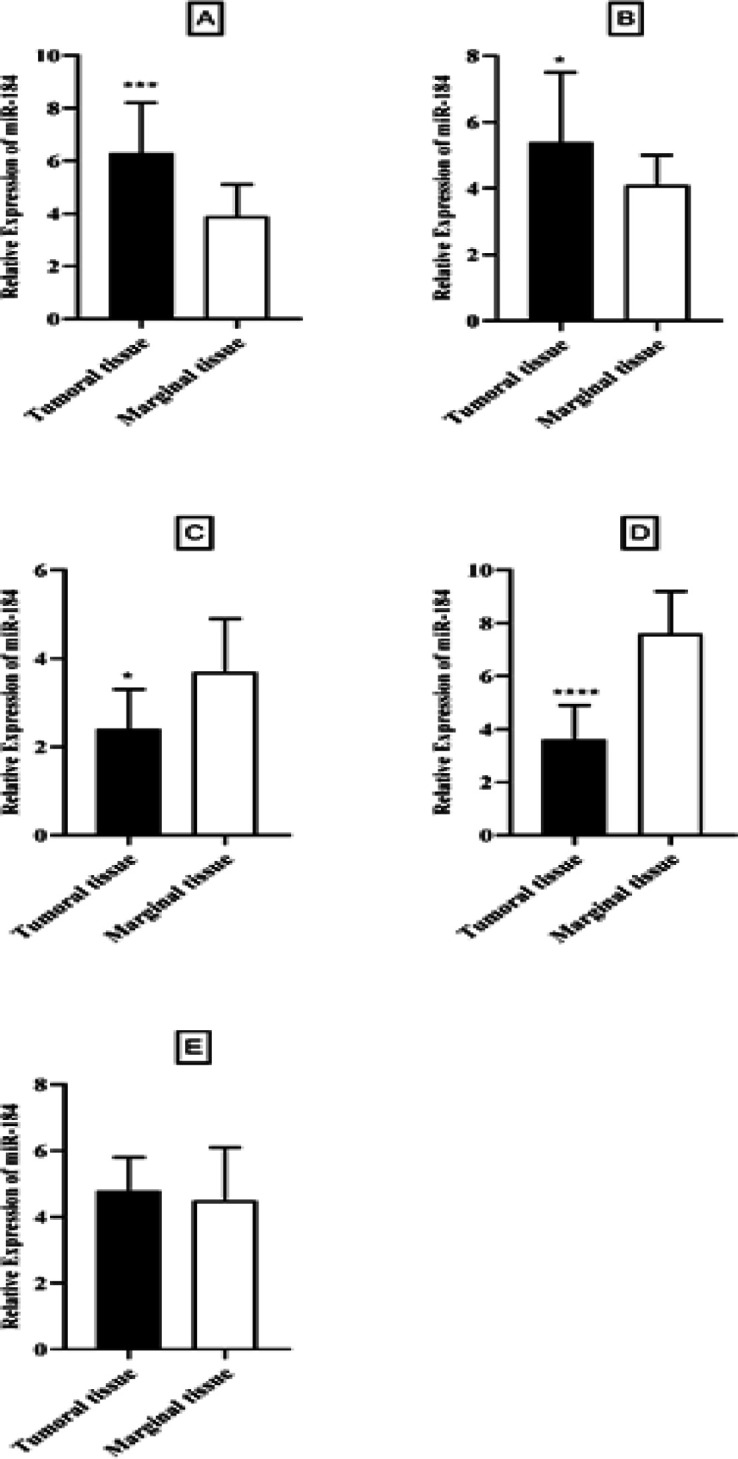
Expression Level of miRNAs in Tumoral Tissues Compared with Marginal Tissues (* shows P<0.05, *** shows P<0.001, **** shows P<0.0001).

**Figure 2.**
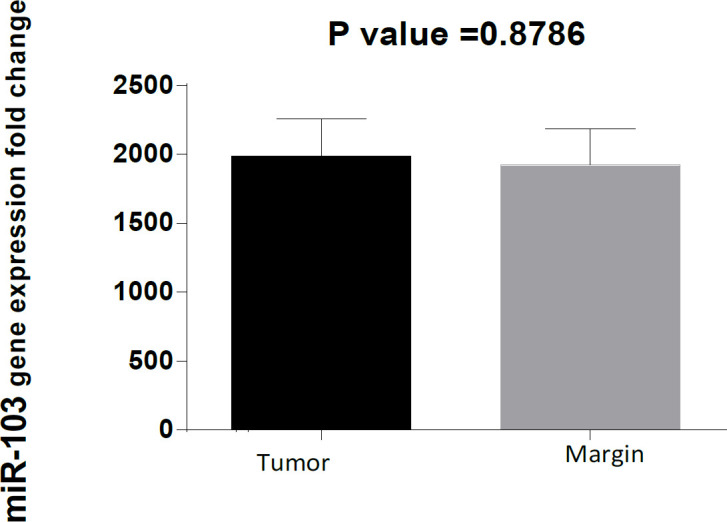
Expression Level of miR-103 in OSCC Cancer Tissue Comparison to Peripheral Ttissue

On the other hand, expression of miRNA-103 did not show any different in comparison with peripheral tumor-free tissues (p < 0.8786, shown in Figure 1). *MiR-184* and *miR-378* have been overexpressed in samples of patient with tongue cancer (P<0.005; Figure 2 and P<0.02; Figure 4 respectively). By contrast, miR497 and miR506 have been downregulated in patient with confirmed tongue cancer compared to peripheral noncancerous tissues (P< 0.043; Figure 5 and P<0.0001; Figure 6 respectively). 

**Table 1:** Clinical Parameter in Tumor Tissues of Patients with Cancer

Characteristic	Classification	Value
Age	<55	20 (40%)
	<55	30 (60%)
Sex	Female	21 (42%)
	Male	29 (58%)
Lymph node metastases	Positive	19 (368%)
	Negative	31 (62%)
Differentiation	Good	19 (38%)
	Moderate	18 (36%)
	Poor	13 (26%)
Venous invasion	Positive	18 (40%)
	Negative	32 (40%)

**Table 2:** Statistical Evaluation of Mirnas Expression in Clinical Samples

MiRNA	P value	Expression
miR103	0.8786	-
miR184	0.005	increase
miR378	0.02	increase
miR497	0.043	decrease
miR506	0.0001	decrease

**Figure 3:**
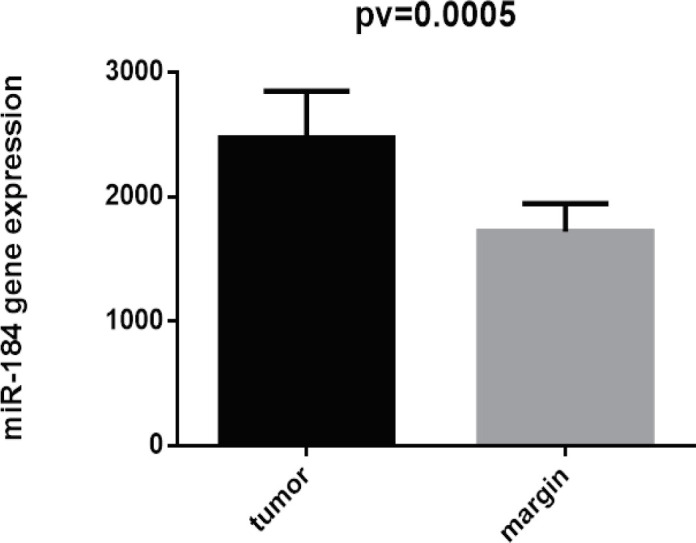
Expression of miR-184 in OSCC Cancer Samples in Comparison with Non-Cancerous Samples

**Figure 4:**
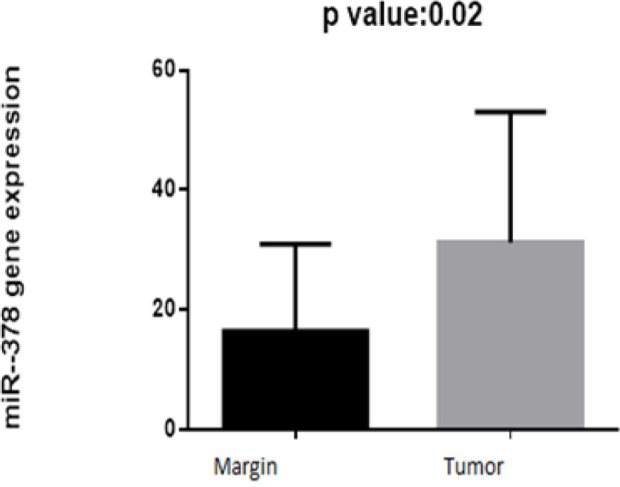
Expression of miR 378 in OSCC Cancer and Cancer Adjacent Normal Tissues

**Figure 5:**
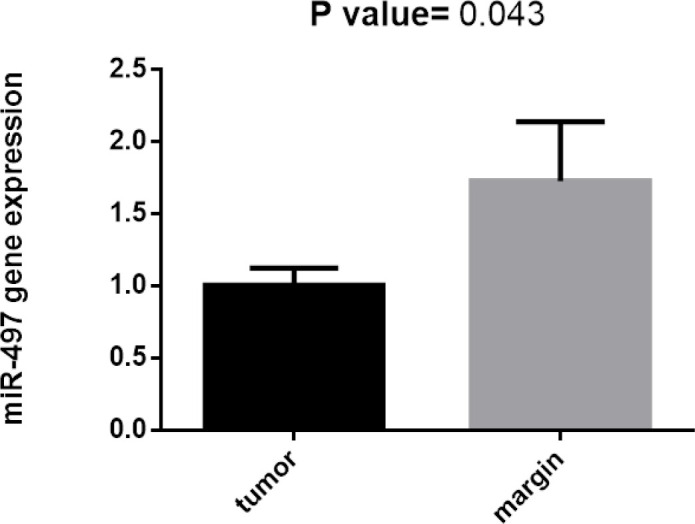
Expression Level of miR-497 in OSCC Cancer Tissue and Marginal Tissue

**Figure 6.**
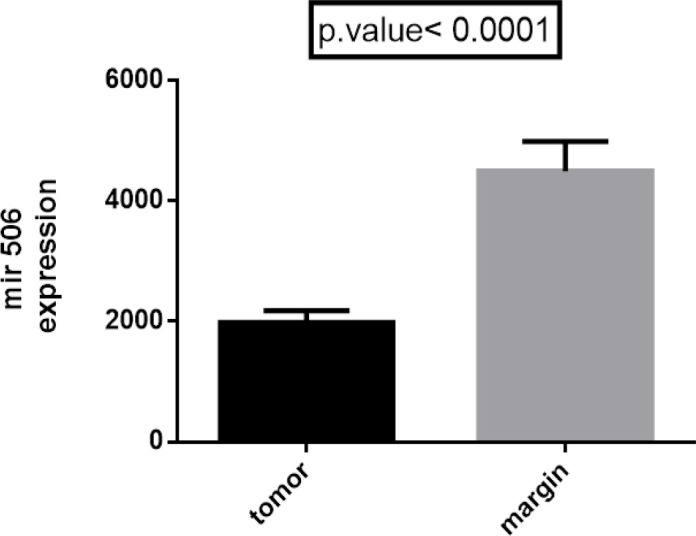
Expression of miR-506 in OSCC Cancer Samples Compared to Non-Cancerous Samples

## Discussion

Accumulating evidence has demonstrated that miRNAs can be analyzed as a diagnostic biomarker and therapeutic tool in various cancers (Asadi et al., 2018a; Asadi et al., 2018c). Moreover, significant effect of miRNAs on gene expression is identified by content of miRNAs, leading to different clinical properties of cancer (Zarredar et al., 2018; Zeng et al., 2018). Therefore, miRNAs have the potential to develop tumor biomarker, predicting prognosis and therapeutic efficacy (Zheng et al., 2017; Shirafkan et al., 2019). Among various miRNAs dysregulated in OSCC* miR-103*, *miR-184*,* miR-375*, *miR-497*, and *miR-506*; are several important miRNAs that contribute to the development and progression of OSCC. MiR 184, as an oncogene in certain types of cancer including squamous cell carcinoma of the tongue, play significant role in antiapoptotic, invasion, and cell proliferation processes. Presence of primary tumor is considered as the most important reason for high plasma expression level of *miRNA-184*, which can be regarded as an innovative cancer marker in tongue squamous cell carcinoma (Wong et al., 2008; Cheng et al., 2015). However, *miR 184* overexpression in plasma was obviously repressed through surgical removal of primary squamous cell carcinoma of tongue (Wu et al., 2017). MiR-497 was first identified as a tumor-related miRNA in breast, colorectal, and lung cancer. Inhibiting cell growth, increasing apoptosis and G0/G1 phase arrest can be noticed as a result of miR-497 upregulation, consequently, convert this miRNA to a potential therapeutic target for the treatment of breast cancer (Hu et al., 2016). On the other hand, abnormality in miR-497 regulation is responsible for clinicopathological properties and overall survival time of the patient in several types of cancers; so that, high regulation level of miR-497 leads to longer overall survival and OS time in comparison with patient with low expression level. Thus, miR-497 could be very substantial biomarker to predict prognosis in patient with cancer patient (Yang et al., 2016). MiRNA-506 is a tumor repressive miRNA, which is downregulated in different types of cancers including hepatocellular carcinoma, cervical cancer, ovarian cancer, and OSCC. Furthermore, miR-506 caused cell cycle arrest at the G1/S transition, and enhanced apoptosis and chemosensitivity of cancer cells (Liu et al., 2015). This study evaluated *miR-103*, *miR-184*, *miR-378*, *miR-497* and* miR-506* expression in 20 patients with tongue cancer samples and 20 paired noncancerous samples by real-time PCR and was calculated using the 2^(-ΔΔCt)^ method. The expression of *miR-103* did not show any significant alteration in cancer samples compared to non-cancerous samples. Whereas, based on obtained results* miR-184* and *miR-378 *were significantly upregulated in tongue tissues in comparison with marginal matched normal tissues. It is worth to mention that, our results proved that expression of *miR497 *and *miR506* represented remarkable decrease in cancerous tissues, while the expression of these two miRNAs was high in cancer adjacent normal tissues. 

In conclusion, the expression of* miR-184* and *miR-378 *are upregulated and expression of miR497 and miR506 are downregulated, and *miR-103* remains unchanged in OSCC tumor tissues. Therefore, changed miRNAs may be as indicators for diagnosis of patients with OSCC and show critical empirical investigative standards.

## Statement conflict of Interest

The authors declare that they have no conflict of interest to report.
